# Effects of interferon-alpha on hippocampal neurogenesis and behavior in common marmosets

**DOI:** 10.1186/s13041-020-00639-9

**Published:** 2020-06-26

**Authors:** Naoko Kaneko, Sayuri Nakamura, Kazunobu Sawamoto

**Affiliations:** 1 Department of Developmental and Regenerative Neurobiology, Institute of Brain Science, Nagoya City University Graduate School of Medical Sciences, 1 Kawasumi, Mizuho-cho, Mizuho-ku, Nagoya, Aichi 467-8601 Japan; 2 Division of Neural Development and Regeneration, National Institute for Physiological Sciences, 5-1 Higashiyama, Myodaiji, Okazaki, Aichi 444-8787 Japan

**Keywords:** Adult neurogenesis, Common marmoset, Dentate gyrus, Depression, Interferon-alpha, Primate, Proliferation

## Abstract

In many mammalian species, the production of new neurons in the hippocampal dentate gyrus continues throughout life. Previous studies using rodents suggest that adult-born neurons are involved in memory and cognition tasks and mood regulation. Interferon-alpha (IFNα), a proinflammatory cytokine used for the treatment of chronic viral hepatitis and malignancies, frequently causes depressive symptoms in patients and animals, including non-human primates. We have previously demonstrated that chronic IFNα treatment decreases hippocampal neurogenesis in mice. Here, we investigated the effects of four-week human pegylated IFNα treatment on hippocampal neurogenesis and behavior in common marmosets. Continuous monitoring of voluntary activity levels using an actigraphy device suggested that adaptive ability is impaired in IFNα-treated animals. Analyses of BrdU-labeled cells expressing a marker for immature or mature neurons revealed a significant reduction in the number of new neurons in the hippocampus of IFNα-treated animals. These data indicate that chronic human IFNα treatment causes behavioral changes and a decrease in hippocampal neurogenesis in common marmosets.

## Main text

In many mammalian species, neural stem cells in the hippocampal dentate gyrus continuously produce new neurons throughout life. These new neurons, which possess distinct electrophysiological properties from those of pre-existing neurons, contribute to hippocampal-dependent memory and cognition and have been implicated in stress responses and depressive behaviors in rodents [1]. The degree of adult neurogenesis in the adult human brain is controversial [2, 3], largely because of ethical and methodological limitations of human studies. Studies using non-human primates, whose brains have structural and functional similarities with the human brain, should provide useful information for understanding the mechanisms and functions of hippocampal neurogenesis in primates.

Interferon-alpha (IFNα) has been used for the treatment of chronic viral hepatitis and several malignancies. However, it causes depression in about 30% of all treated patients, which frequently prevents the completion of treatment [4]. Furthermore, IFNα treatment also induces depression-like behavioral changes in rodents [5–7] and non-human primates [8, 9]. Impaired monoamine signaling and inflammatory responses are involved in IFNα-induced depression, although their precise mechanisms are still unclear. We have previously reported that IFNα-treatment decreases hippocampal neurogenesis and induces depression-like behavioral changes in mice via type-1 IFN receptors in the central nervous system [5]. IFNα also reduces neurogenesis of human hippocampal neural progenitors in vitro [10]. Here, we investigated the effects of chronic IFNα treatment on behavior and hippocampal neurogenesis in common marmosets (*Callithrix jacchus*), which are small-bodied monkeys established as laboratory animals for preclinical research.

**Fig. 1:**
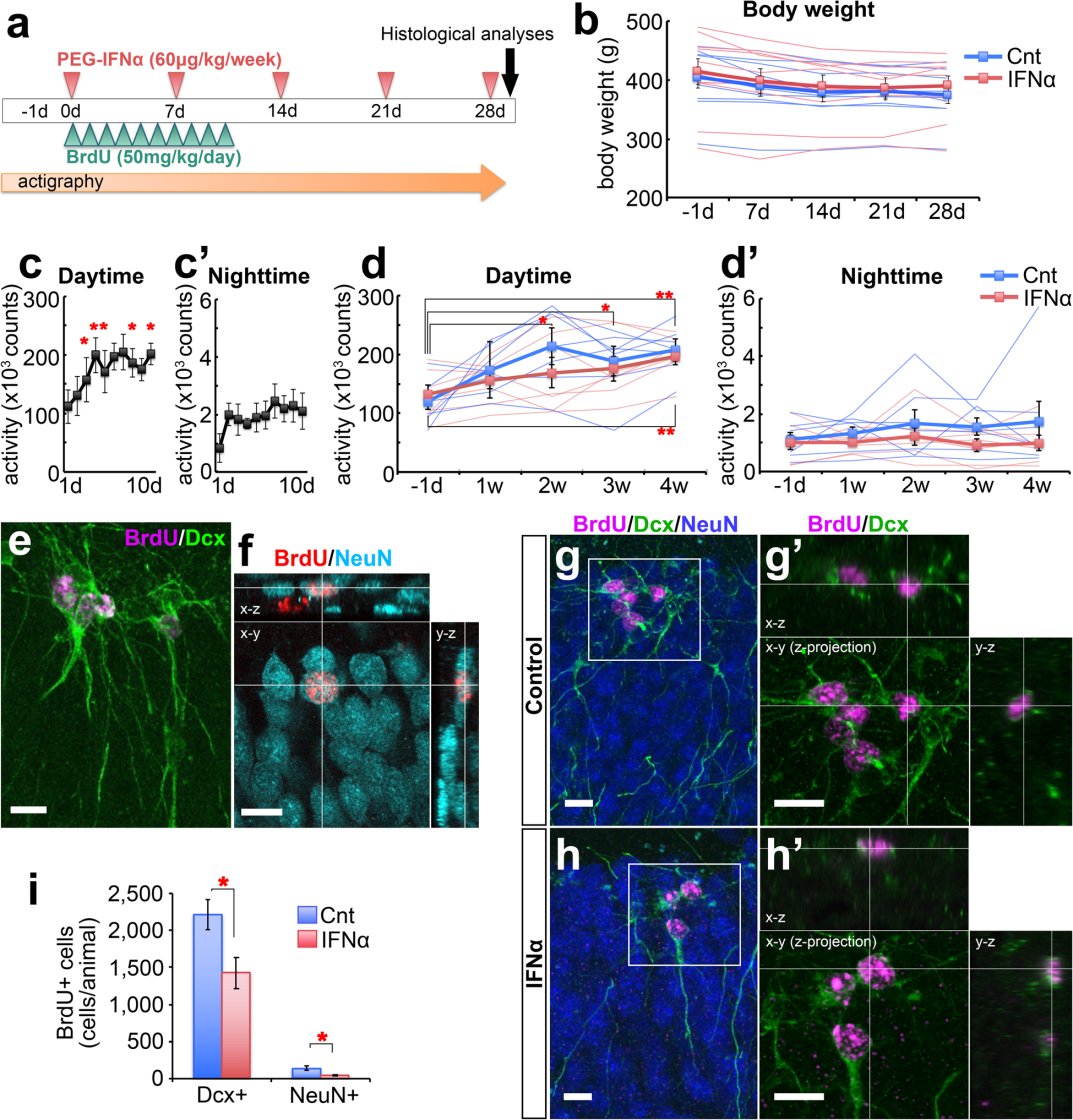
Effects of IFNα on behavioral activity and hippocampal neurogenesis in common marmosets. **a:** Experimental procedures. Adult common marmosets were treated with human pegylated IFNα or vehicle (control, Cnt) once a week for 4 weeks (see Materials and Methods in supplemental information), and BrdU was injected once a day for the first 10 consecutive days. The voluntary activity of each animal was continuously monitored by actigraphy from the day before (− 1d) treatment to the end of the experimental period. The animal tissues were fixed at day 28 for histological analyses. **b:** Mean bodyweight of the animals in the control and IFNα-treated groups before (on day − 1) and during drug administration (on day 7, 14, 21, and 28). **c-c’:** Daytime (c) and nighttime (c’) mean activity counts of animals for 10 days after fitting the actigraphy device. **d-d’:** Daytime (d) and nighttime (d’) mean activity counts of animals a day before (−1d) and each week during IFNα or vehicle treatment. **e-f:** Representative images of BrdU-labeled (BrdU+) cells in the SGZ and GCL of the dentate gyrus. The Z-stack projection image shows BrdU+ cells (magenta) expressing Dcx (green) (e). Confocal image showing a BrdU+ cell (red) expressing NeuN (cyan) (f). The x-z and y-z planes are presented in the top and right panels, respectively. **g-h:** Z-stack projection images of the dentate gyrus immunostained for BrdU (magenta), Dcx (green), and NeuN (blue) in control (g) and IFNα-treated (h) animals. High-magnification images of the boxed areas in (g) and (h) are shown in (g’) and (h’), respectively (BrdU: magenta, Dcx: green, The x-z and y-z planes are presented in the top and right panels, respectively). **i:** The number of BrdU+ cells that express Dcx or NeuN in the dentate gyrus (SGZ and GCL). Scale bars: 10 μm (e-h’). The quantitative data are presented as the mean ± SEM. *P < 0.05, **P < 0.01

Considering clinical protocols, we subcutaneously injected human pegylated IFNα or vehicle once a week for four weeks into young-adult male and female common marmosets (Fig. 1a, Additional file 1). To label newly-generated neurons, BrdU (50 mg/kg/day) was intraperitoneally administrated once a day for ten consecutive days from the day of the first IFNα treatment. The body weights showed no significant difference at any time point between the vehicle-treated (control) and IFNα-treated groups (Fig. 1b). The voluntary activity of each animal was continuously monitored with a small actigraphy device. A pilot study without drug injections showed that the daytime activity levels gradually increased after the device was fitted and reached a plateau within several days due to adaptation (Fig. 1c-c’). Therefore, we fitted the device two days before the first drug administration. However, repeated injections in control animals delayed adaptation, leading to increases in daytime activity in the second week and thereafter (Fig. 1d). IFNα-treated animals, however, showed no such increases until the third week (Fig. 1d), suggesting that their adaptive ability was impaired. These data also suggest that IFNα decreased daytime activity, similar to symptoms observed in patients, although we did not detect any statistical significance because of large inter-individual variance. IFNα treatment frequently causes insomnia in patients; however, the IFNα-treated marmosets did not show statistically-significant alterations in nighttime activity (Fig. 1d’), possibly due to the differences in sleep patterns among primates [11].

We next examined the distribution of BrdU-labeled (BrdU+) cells in the subgranular zone (SGZ), where neural stem/progenitor cells reside, and granular cell layer (GCL), the destination of newly-generated neurons, of the hippocampal dentate gyrus. Most BrdU+ cells were located in the inner layer of the GCL and expressed the immature neuronal marker, doublecortin (Dcx) (Fig. 1e). BrdU+ cells expressing the mature neuronal marker, neuronal nuclei (NeuN), were observed only at low frequency (Fig. 1f) because these neurons take longer to mature in primates compared with those in the rodent GCL [12, 13]. The numbers of BrdU+Dcx + cells and BrdU+NeuN+ cells in these areas were significantly decreased in the IFNα-treated group compared with those in the control group (Fig. 1g-i). Taken together, IFNα treatment significantly suppressed behavioral activity and diminished hippocampal neurogenesis in common marmosets, consistent with our previous studies using rodents [5, 6].

To analyze the impact on slow neuronal maturation in primates more precisely, a longer IFNα treatment is needed. However, we did not extend the treatment period because repeated injections of human IFNα in common marmosets can lead to the production of antibodies that neutralize its biological activity. Therefore, it is likely that the IFNα-induced behavioral changes observed in this study did not result from decreased neurogenesis, but were associated with other effects such as acute inflammation. However, we cannot exclude the possibility that adult-born immature neurons play some role in hippocampal function in common marmosets. Given the differences in spatiotemporal distribution and biological properties of adult-born hippocampal neurons between rodents and primates [13, 14], their involvement in mood regulation may also be different. Furthermore, physical exercise promotes hippocampal neurogenesis [15]; therefore, it is also possible that the higher level of neurogenesis in the control animals compared with IFNα-treated animals might be caused by their earlier increase in voluntary activity during the experimental period (Fig. 1d). Further studies using common marmosets will lead to a better understanding of the effects of IFNα on mood and neurogenesis in primates.

## Supplementary information

**Additional file 1.** Materials and Methods.

## Data Availability

All data required to evaluate the conclusions presented in this study are included in the manuscript or its supplementary information file.

## Abbreviations

IFNα: Interferon-alpha
Dcx: Doublecortin
NeuN: Neuronal nuclei
SGZ: Subgranular zone
GCL: Granule cell layer
