# Supplementary material for: Effects of interferon-alpha on hippocampal neurogenesis and behavior in common marmosets
Source: Mol Brain. 2020 Jun 26;13:98. doi: 10.1186/s13041-020-00639-9 (PMC7318457; doi:10.1186/s13041-020-00639-9)
Supplement: Supplementary file 1 — Additional file 1. Materials and Methods. [file 13041_2020_639_MOESM1_ESM.docx]

**Materials and methods**

**Animals**

Young adult (control: 1.79±0.14-year-old, IFNα: 1.76±0.11-year-old) male (*n*=8) and female (*n*=11) common marmosets (*Callithrix jacchus*) obtained from three mating pairs in a domestic animal colony in Nagoya City University were used. The animals were housed individually with water and food *ad libitum* in a temperature-controlled room (24±1°C) with a 12 h light-dark cycle. All experiments using live animals were performed in accordance with the guidelines and regulations of Nagoya City University.

**Activity monitoring**

Some of the animals were fitted with a custom-made jacket equipped with a small actigraphy device (Actiwatch, Bioresearch Center, Aichi, Japan) two days before the first drug administration to continuously record their physical activity throughout the experimental period. Due to the substantial inter-individual differences in voluntary activity recorded by actigraphy, the average activity counts of each animal during each week were compared with those recorded one day before IFNα treatment.

**IFNα and BrdU treatment**

The animals were subcutaneously injected with human pegylated IFNα (PEGASYS, Roche, Basel, Switzerland, 60 μg/kg/week) or vehicle (0.1 M sodium acetate buffer, pH 6.0) once a week for 4 weeks. The binding of IFNα to its receptors is species-specific (1,2); therefore, we determined the dose required for our study was about 20-fold higher than that used in clinical applications.

To label newly-generated neurons, BrdU {Millipore Sigma, Burlington, MA, USA, 50 mg/kg/day, dissolved in sterile phosphate buffer saline (PBS)} was intraperitoneally administrated once a day for ten consecutive days from the first day of IFNα treatment.

**Tissue processing**

The animals were deeply anesthetized with isoflurane and fixed by transcardial perfusion with 150 mL PBS (pH 7.4), followed by 300 mL 4% paraformaldehyde (PFA) in 0.1 M phosphate buffer (PB, pH 7.4) for immunohistochemistry 2–4 h after the last (fifth) IFNα treatment. After overnight post-fixation in 4% PFA, 60-μm-thick coronal brain slices were prepared using a vibratome, then stored in 0.04% sodium azide-containing PBS at 4°C until use.

**Immunohistochemistry**

Every 12th coronal brain section was incubated for 1 h in blocking solution (10% donkey serum and 0.4% Triton X-100 in PBS) and then for 36–48 h at 4°C with a rabbit anti-Dcx antibody (1:200, Cell Signaling Technology, Danvers, MA, USA) and a mouse anti-NeuN antibody (1:100, Millipore Sigma, MAB377). After the sections were washed, they were incubated for 3 h at room temperature with Alexa Fluor-conjugated secondary antibodies (1:1,000, Life Technologies, Carlsbad, CA, USA), then fixed with 4% PFA for 30 min. For BrdU staining, sections were treated with 1 M HCl at 64°C for 45 min and 1% H_2_O_2_ for 1 hour. Following incubation in a blocking solution for 1 h, sections were incubated with a rat anti-BrdU antibody (1:200, Abcam, ab6326, Cambridge, UK) for 48 h at 4°C. After incubation with primary antibodies, biotinylated anti-rat IgG secondary antibodies (1:1,000, Jackson Laboratory, Bar Harbor, ME, USA), a Vectastain Elite ABC kit (Vector Laboratories, Burlingame, CA, USA), and a TSA Fluorescence System (PerkinElmer, Waltham, MA, USA) were used to visualize staining.

**Quantification**

Cell numbers were quantified using every 12th section under a confocal laser microscope (LSM700 and LSM880, Carl Zeiss, Jena, Germany). The counted cell numbers were multiplied by 12 to represent cells per animal.

**Statistics**

All data are expressed as the mean ± standard error of the mean (SEM). Normality and equal variances between group samples were assessed using the Shapiro-Wilk test and *F* test, respectively. When normality and equal variance between sample groups were achieved, differences between means were determined using a two-tailed Student’s *t-*test and a paired *t-*test. A *P* value of <0.05 was considered to be statistically significant.

**References**

1. Veomett MJ, Veomett GE. Species specificity of interferon action: maintenance and establishment of the antiviral state in the presence of a heterospecific nucleus. J Virol. 1979;31(3):785–94.

2. Uhlendorf CP, Zimmerman EM, Baron S. Heterologous Activity of Monkey Interferons. Proc Soc Exp Biol Med. 1973;144(2):628–32.
